# Ensemble machine learning framework with SHAP and LIME for accurate early prediction of student success in online learning environments

**DOI:** 10.1038/s41598-026-49894-1

**Published:** 2026-04-28

**Authors:** Essa E. Almazroei

**Affiliations:** Learning Design and Technology Department, College of Education, University of Jeddah, Jeddah, Saudi Arabia

**Keywords:** Learning analytics, Student success, Ensemble machine learning, Predictive model, Explainable artificial intelligence, Computational biology and bioinformatics, Mathematics and computing

## Abstract

The development of digital learning environments has generated rich educational data capable of supporting early prediction of student outcomes. In this study, seven diverse datasets, spanning demographics, parental education, assessment history, and VLE engagement, were integrated into a unified machine-learning pipeline. Four supervised algorithms (Logistic Regression, Random Forest, XGBoost, and Multi-Layer Perceptron) were trained using SMOTE and stratified fivefold cross-validation. XGBoost demonstrates the strongest performance among the evaluated models, achieving 95.04% accuracy, ROC-AUC of 0.9879, F1-score of 0.95, precision of 0.96, and recall of 0.95 on the test set. Cross-validation further confirmed the model’s robustness (mean ROC–AUC = 0.9879). The Bagging ensemble also demonstrated competitive performance, achieving a cross-validation accuracy of 0.9487, a cross-validation F1-score of 0.9470, and a test accuracy of 0.9504. To ensure interpretability, SHAP and LIME were employed, revealing cumulative assessment performance, withdrawal patterns, and engagement intensity as the most influential predictors. The results demonstrate that combining strong predictive algorithms with explainable AI (XA) creates a reliable early-warning system capable of supporting targeted academic interventions and improving institutional decision-making.

## Introduction

The digital learning environment has drastically changed the way students access scholarly materials, teaching personnel, and institutional services. Learning Management Systems^1^ (LMS), Virtual Learning Environments (VLEs), Web-based assessment^2^, and electronic registration systems^3^ have now become everything that accompanies a learner throughout their educational journey. The ongoing generation of behavioral, temporal, and performance data has placed Learning Analytics (LA)^4^ as the key tool in explaining and improving student performance. As a result, higher education institutions worldwide increasingly rely on this empirically informed understanding to predict the risk of academics, improve pedagogical strategies, and launch early responses that strengthen the retention and advancement of students.

Although this is a possibility, the development of well-trusted predictive models^5^ of student success is a complex issue. The outcome of academic results is a complex interaction of demographic features, socioeconomic predictors, engagement patterns, previous performance, and time behaviors, including registration delays or dropout incidents. Current risk-prediction models^6^ often focus on only a small number of these signals or do not suit the level of noise and class imbalance that exists in learning data. Furthermore, numerous machine-learning models are not interpretable, and thus, they cannot be used in education where transparency, accountability, and fairness are crucial^7^.

In order to address these shortcomings, this research work suggests a detailed predictive model that combines seven interlinked datasets, namely: learner demographics, module enrolments, assessment submissions, engagement traces, and course-level metadata to predict academic outcomes with high accuracy and interpretability. The proposed model considers four supervised learning algorithms, namely, Logistic Regression^8^, Random Forest^9^, XGBoost^10^, and Multilayer Perceptron^11^, within a unified pipeline that standardizes preprocessing, provides class imbalance by using SMOTE^12^, and demonstrates robust generalization by using stratified fivefold cross-validation. A copilot Bagging ensemble^13^ is also proposed to determine whether aggregated decision structures can further increase the power of prediction.

Although model accuracy is an extremely critical measure, the use of predictive systems in education requires the availability of clear reasons behind every prediction. Based on this, the paper uses the latest Explainable Artificial Intelligence (XAI)^14^ methods, namely SHAP^15^ and LIME (igoche^16^), which are designed to be globally and locally interpretable and instance-explainable, respectively. These methodologies shed light on the inner logic of the best model, and which behavioral schemes, academic indicators, and time qualities have the most significant effect on the forecasts of student performance.

This study presents a framework of scalable and understandable early identification of at-risk learners by combining strict predictive modelling with clear explanatory methodologies. The results indicate how sophisticated machine learning, with the aid of an integrated and explainable feature set, can provide institutions with applicable insights to inform personalized academic support and boost student achievements in large-scale digital learning settings^17^.

## Problem statement

Online learning has brought about large volumes of behavioral, demographic, and academic data^18^, but the challenge of combining these heterogeneous streams into reliable predictors of student success has been difficult. The existing models often depend on a limited or backward-looking indicator, thus limiting the early detection of vulnerable learners and reducing the effectiveness of timely response. Further, learning data are highly dimensional, and most modelling methods cannot represent them with their nonlinear, imbalanced, and time-related complexities, leading to poor prediction performance. Poor interpretability of even the most performant machine-based models has made them rarely used in higher-education settings because of reduced trust, transparency, and practical recommendations to teachers.

In this way, the proposed research work will integrate controlled machine-learning algorithms^19^ and sophisticated data pre-processing, imbalance reduction, and model-independent interpretability techniques (SHAP and LIME). The aim is to build a clear and trustworthy system of prediction^20^, which supports proactive, evidence-based academic advising in mass online learning.

## Objective of the study

The general purpose of this study is to build an analytically rigorous and interpretable predictive modelling framework that will be able to detect students who are at risk of performing academically below expectations in the large-scale digital learning systems. In the achievement of this goal, the study will follow the following objectives:

- To aggregate and pre-process multi-source learning data^21^, such as demographics, test scores, course schedules, and VLE interaction logs, to generate a single feature space, which can be analyzed by machine learning.
- To create and test several supervised learning algorithms (Logistic Regression, Random Forest, XGBoost, and Multilayer Perceptron) with stratified cross-validation, thus guaranteeing the high-quality estimates of model performance, considering even the imbalance in classes.
- To infer the use of ensemble learning (Bagging)^22^ and establish whether aggregated prediction schemes can produce improvement in accuracy, stability, and generalization over and above the performance of individual classifiers.
- In order to introduce SMOTE-based oversampling strategies to balance the unequal distribution of academic performance and, therefore, enhance sensitivity to minority (at-risk) students.
- To use SHAP and LIME interpretability techniques to produce transparent, model-agnostic explanations that can be used both globally and individually to allow educators and analysts to interpret the reasons behind one or the other of the following: success or risk.
- To determine the relevance of predictive insights in practice in academic advising, early warning interventions, and policy development within institutions.

## Literature review

This systematic literature review explores the application of predictive modelling in educational settings, focusing on the Open University Learning Analytics Dataset (OULAD)^23^ from 2017 to 2025. The review synthesizes key findings from seventeen studies, highlighting the prevalent use of machine learning and deep learning models to predict student performance, identify at-risk learners, and forecast engagement. Despite advancements, challenges such as model complexity, interpretability, and data quality persist, with a growing emphasis on the need for more explainable and empirically validated approaches.

This paper^24^ presents an online incremental learning system that is aimed at predicting student performance based on continuous streams of data that are characteristic of Massive Open Online Courses (MOOCs). As opposed to the traditional batch-based models, where the models are based on the pre-collected data, the incremental approach is updated in real time as new student data becomes available. Such a methodology is supported by a genetic algorithm, which builds balanced, diverse training subsets taking into consideration memory constraints. Comparative studies show that a genetic-algorithm-based strategy has better stability of the model and accuracy of the models, and the variability of performance is less as opposed to random sampling approaches. When applied to the OULAD data, the method provides high predictive accuracy with reduced standard deviation in comparison to the current methodological approaches. An incremental model is very beneficial, especially with real-time applications within online education systems to enable timely pedagogical interventions.

The authors of this study^25^ developed a proactive monitoring system, which is based on data analytics to evaluate the performance of students at the beginning of the academic year and to provide individualized instruction interventions. The framework combines machine-learning versions, i.e., Random Forest, XGBoost, and Deep Neural Networks, with Explainable AI (XAI) architectures to generate interpretable forecasts. Using the corpus of OULAD, the platform can identify at-risk learners in the first four weeks, and this would facilitate adaptive testing in time and curated study materials. Early assessments show that the overall accuracy is more than 85 per cent, with a 15-20 per cent engagement and passing rates as well. The authors note the relevance and significance of real-time, open predictive analytics in improving scholastic performance and reducing attrition to provide a fresh paradigm of early support interventions.

The paper is a descriptive study^26^, which gives a detailed analysis of the phenomenon of dropouts in the OULAD database. It explores the combined effect of demographic factors, degree of engagement, and academic achievement on the disengagement propensity of students. The main indicators, such as age, academic discipline, average grades of assessment, and the use of virtual learning environments (VLEs), are measured in a systematic manner in order to identify the trends that reflect signs of disengagement. The authors use Python for data cleaning and Power BI for visualization, which gives an in-depth tableau of established causes of dropout. Though no predictive model is put forward, the results shed light on vital tendencies that teachers and administrators can use to mitigate the possibility of dropout and reinforce retention in distance-learning settings.

The paper^21,27^ evaluates Federated Learning (FL) as a privacy-friendly alternative to predicting at-risk students based on the OULAD repository. The authors contrast FL with the traditional centralized methods, which include Logistic Regression and Deep Neural Networks, where the authors raise questions on issues related to do with the confidentiality of the data. Studies are done on the predictive performance of models in terms of model complexity and local data balancing. Empirical findings indicate that the federated strategy can deliver a healthy ROC AUC of about 85% percent. The conclusion is made that FL provides a scalable privacy-focused solution that can be used to deploy early-warning systems without accessing personal data of students, which confirms its potential in the educational field without violating regulatory requirements.

This study^28^ examines the perspectives of postgraduate data-science students on learning analytics by surveying them on the topic of learning analytics, including a mixed-method approach of human-directed content analysis and large language model (LLM) inference. Participant contributions are analyzed in the OULAD context, and their conceptual understanding of learning analytics, as well as the pedagogical consequences thereof, is evaluated. Themes that are considered to be core and that appear are the importance of predictive analytics and the application of various machine-learning methods to improve educational performance. The findings indicate that students of data science have a high level of knowledge about learning analytics, thus contributing to the discussion of how the perspectives of learners can be used to design and implement the analytics systems that would address their particular needs and realities of the context.

The article^29^ suggests an integrative approach, in which Long Short-Term Memory (LSTM) networks are combined with SHapley Additive Explanations (SHAP) to predict academic performance of physically or cognitively disabled students in Virtual Learning Environments (VLEs) contexts. The research employs the OULAD data, which helps to deal with the peculiarities of disabled students, such as unusual learning patterns and the lack of accessibility. The LSTM component captures sequential relationships inherent in a record of interactions, and SHAP attribution gives explicit explanations by isolating features of impact on performance results. The hybrid system has achieved an accuracy of 92.88 , which is even higher than other conventional systems, which include the Random Forest and XGBoost. The results indicate the effectiveness of deep learning with explainable AI to provide targeted and understandable interventions to disabled learners.

The paper^30^ is a comparative study of a range of machine-learning (ML) and deep-learning (DL) models to predict student success in Massive Open Online Courses (MOOCs) using the OULAD data source. Applied models include Logistic Regression, Decision Trees, Random Forest, and Support Vector machines, and deep networks, including Convolutional Neural Networks, Recurrent Neural Networks, and Long Short-term Memory networks. The findings show that LSTM networks give higher accuracy and precision with reported rates of 83.41 and 82.20, respectively. The paper goes further to explain the benefits of DL methods in capturing complex patterns across the engagement data, thus giving meaningful actions that can be used to conduct informational instructional design and student-specific interventions.

This paper^29,31^ presents a bidirectional long short-term memory (Bi-LSTM) model, specifically designed to predict student academic success, and, in specific detail, to provide an overall understanding of the decision process with the help of SHapley Additive Explanations (SHAP). With the use of the Open University Learning Analytics Dataset (OULAD) it was found through the Bi-LSTM model that there were second-term grade-point averages (GPAs) that were accurate with 88.23% accuracy. This is better than the traditional machine-learning classifiers, such as CatBoost, XGBoost, and LightGBM. This is because the SHAP values added provide greater interpretability, as it is possible to explicitly identify the salient features having an impact on academic outcomes. The model was statistically validated using analysis of variance (ANOVA) and Friedman tests, and both of them supported the superiority of the model in comparison with other methods. On the whole, the results support the effectiveness of deep-learning techniques in the field of educational data mining and highlight the opportunity to forecast student achievement and reduce the risk of dropout.

The article^19^ explores the implementation of Federated Learning (FL) to predict students considered at-risk in online instruction, which is based on the OULAD dataset, and compares FL to conventional machine learning paradigms. It investigates the trade-offs between the complexity of the model and the necessity of data privacy. At the core of this motivation would remain student confidentiality across institutional borders. The authors hypothesize that FL can achieve competitive predictive accuracy without compromising on privacy, as seen in varying distributed organizations. The article asserts that FL is a scalable, secure paradigm that can be used in education systems to detect and assist vulnerable students according to the existing data-protection policies. The summary of some previous research work is shown in Table 1 below:

**Table 1:** Summary of predictive models and key findings from previous studies using the Open University Learning Analytics Dataset (OULAD).

References	Model Used	Dataset	Accuracy	Key Findings	Limitations
^24^	Online incremental learning/streaming data	OULAD	N/A	Handling streaming data, memory constraints, and class-balance	Proof of concept only; small sample
^25^	Random Forest, XGBoost, DNN	OULAD	85% +	A proactive system that predicts at-risk students early for intervention	No predictive model for post-intervention outcomes
^26,21^	Descriptive Analysis	OULAD	N/A	Identified trends in dropout based on demographics and VLE engagement	No predictive model
^27^	Federated Learning	OULAD	85%	Federated learning preserves privacy while predicting at-risk students	Requires more computational resources
^28^	ML & LLM Content Analysis	OULAD	N/A	Explores students’ perspectives on learning analytics through content analysis	No direct prediction model was used
^29,31^	LSTM + SHAP	OULAD	92.88%	LSTM model with SHAP for interpretable prediction of academic performance	Limited generalizability to other datasets
^30^	LSTM, CNN, RNN	OULAD	83.41% (LSTM)	Compared machine learning and deep learning models for performance prediction	The LSTM model was the best, but not always consistent
^29^	Bi-LSTM + SHAP	OULAD	88.23%	Bi-LSTM outperforms other models; SHAP is used for explainability	Accuracy varies by model

### Limitations of previous work

There are a number of limitations that appear in the literature surveyed, as shown in Table 1. The most prominent of these is the problem of generalizability; most models perform well on OULAD but fail to perform on other datasets or different educational settings, as it has been discussed in the articles of^29–31^. Another form of major challenge is the quality of the data. Lacks of demographic or engagement variables are potentially disastrous for predictive performance, which was observed in their work^24^. There is also a significant obstacle to interpretability. The obscure character of these models may still reduce the trust educators have in the model and the action they take in line with the generated predictions, which can be seen in the studies by^29^. Computational complexity also restricts practical implementation. As mentioned by^29^, the federated learning design and deep learning architecture can be expensive due to their resource-intensive nature, which makes them infeasible in many institutions with weak technical infrastructure. Furthermore, the apparent lack of empirical validation in a practical educational context makes the practical applicability of suggested models limited. This is the gap that can be observed in the works. Lastly, other studies did not consider the non-academic determinants, including mental health, which narrows the scope of the predictive frameworks, which is a weakness of their study.

### Contributions and novelty of the study

This research work makes a significant contribution to the research of learning analytics and predictive modelling in the higher educational sphere.

### A multi-file, multi-dimensional predictive framework

By combining seven separate educational files that contain demographic, behavioral, assessment, and time-related data, the authors overcome the limitations of the previous literature based on the use of single-source data or observation of restricted sample characteristics of students. The extended feature space consequently provides a granular and holistic model of student learning behavior at the institutional level.

### Systematic comparison of four supervised learning algorithms

The study offers a comparative analysis of the four common machine learning models to be used, including Logistic Regression, Random Forest, XGBoost, and a multilayer perceptron system in a single processing pipeline, which uses the same preprocessing and resampling procedures. This design allows a valid, repeatable measure of the relative strengths and limitations of each model.

### Integration of ensemble learning for enhanced performance

The strategy of bagging ensemble is used to test the hypothesis of whether collaborative decision-making among the base learners can outperform individual models. The obtained results provide new empirical evidence of ensemble efficacy in the context of learning analytics.

### Rigorous handling of imbalanced educational outcomes

The authors can properly address the problem of the presence of an old-fashioned imbalance between successful and at-risk learners by including SMOTE oversampling in each cross-validation fold. The mathematical accuracy of the predictive models increases their fairness and sensitivity.

### Dual-level explainability through SHAP and LIME

The framework combines both global interpretability through SHAP and local interpretability through LIME to provide a complete, end-to-end explanation of the way in which predictions are made. This kind of dual-layered solution is not well studied in the existing body of learning analytics research, and enhances the practical interpretability of the system.

### Action-oriented insights for academic intervention

In addition to the reporting of the numerical measures of performance, the study illustrates how the model outputs can be converted into actions to be applied in academic practice. It determines the main behavioral patterns, important academic pointers, and certain instances when institutional assistance is needed proactively.

### Scalable and reproducible methodology

The authors provide a scalable template that can be used in the future in institutions using publicly documented algorithms, XAI techniques that have been reproduced, and transparent preprocessing pipelines.

## Methodology

**Figure 1:**
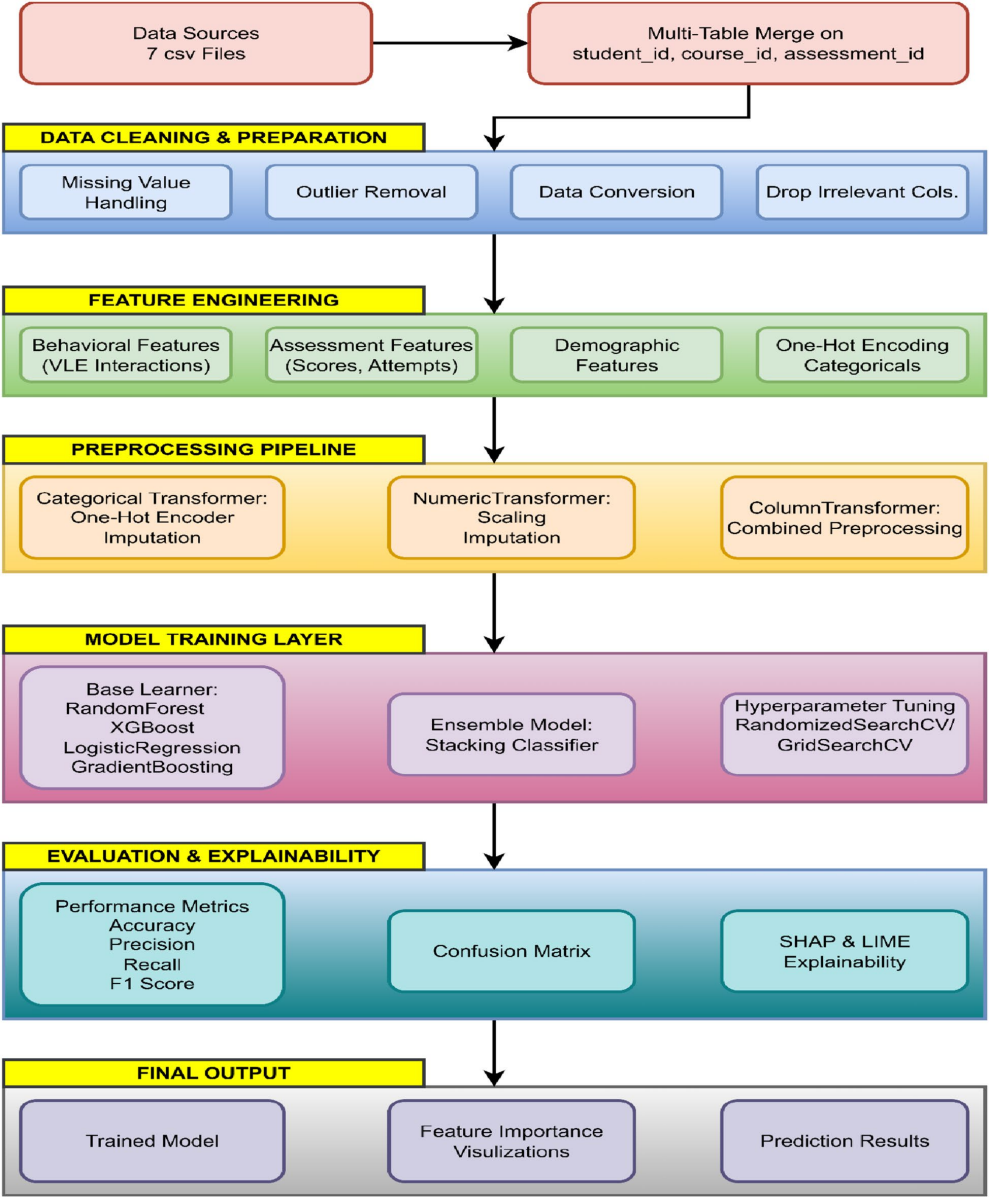
Pipeline architecture for data processing, model training, and evaluation with explainability.

The methodological framework of the study will focus on the development of a reliable predictive system able to identify students who are likely to succeed or face academic difficulties. Multiple data sources were acquired and consolidated, followed by extensive preprocessing, feature construction, model development and evaluation, and analysis for interpretability via state-of-the-art XAI techniques (Fig. 1).

The pipeline begins with data collection from seven CSV files, followed by cleaning, missing value handling, and feature selection. Key features are engineered from behavioral, assessment, and demographic data, with categorical variables one-hot encoded. A combined preprocessing pipeline is used for scaling and imputation. Multiple base models, including Random Forest, XGBoost, and Logistic Regression, are trained, and an ensemble stacking classifier is applied for improved performance. The model is evaluated using metrics like accuracy and precision, with SHAP and LIME ensuring interpretability.

### Dataset overview

The dataset used in this study was obtained from a publicly available source on Kaggle. Each file represents a distinct component of the student learning lifecycle. The details of the dataset are shown in Table 2.

**Table 2:** Description of dataset files.

file name	Records	Key columns	Description
studentInfo.csv	≈ 32,000 records	id_student, code_module, gender, region, highest_education, imd_band, age_band, num_of_prev_attempts, studied_credits, disability, final_result	Provides demographic, educational, and enrollment details for each learner
studentVle.csv	≈ 10–12 million records	id_student, id_site, date, sum_click	Logs daily interactions with the online learning interface, capturing behavioral data
vle.csv	≈ 6000 records	id_site, activity_type, week_from, week_to	Contains descriptive information about available learning resources
studentAssessment.csv	≈ 250,000 records	id_student, id_assessment, date_submitted, score, is_banked	Includes detailed assessment submissions and marks obtained by learners
assessments.csv	≈ 300 records	id_assessment, assessment_type, date, weight	Outlines all assessment components within each module
studentRegistration.csv	≈ 300,000 records	id_student, date_registration, date_unregistration	Tracks module registration timelines and withdrawal events
courses.csv	≈ 100 records	code_module, module_presentation_length	Specifies the academic calendar and duration of module presentations

It is shown in Table 2 that after integration of complete records, the final analytical dataset included about 6500 students and a complete set of 87 engineered explanatory variables, coupled with a binary outcome variable representing academic success.

### System specification description

The model was trained using Google Colab’s cloud-based environment, equipped with an NVIDIA T4 GPU, which provided accelerated computation for model training and optimization. The local system used to manage development and experimentation was a 64-bit machine running on an 11th Generation Intel Core i5-1135G7 processor operating at 2.40 GHz, with 8 GB of installed RAM (7.75 GB usable). The device operated on an x64-based architecture, identified by Device ID A0DCF75D-0F7A-4709-901B-2CB21128EDB8 and Product ID 00329-00000-00003-AA472. This combination of local computational resources and cloud-based GPU acceleration ensured efficient execution of data preprocessing, model training, and evaluation workflows.

### Definition of outcome variable

The target variable was obtained from the final_result attribute. Accordingly, the learners who received a Pass or Distinction were considered academically successful. The ones that received Fail or Withdrawn were considered academically at-risk, respectively. This classification system agrees with early-intervention paradigms in learning analytics, whereby the central goal is to identify the risk well before actual failure occurs.

### Pre-processing procedures

The dataset was strongly refined before the model development. Numerical values missing were imputed using median substitution, and the categorical entries that were missing were substituted using the modal category. One-hot encoding was used to encode categorical predictors so that they could be used in machine learning algorithms. Due to class imbalance, i.e., the successful students significantly outnumbered at-risk learners, the Synthetic Minority Over-sampling Technique (SMOTE) was directly integrated into the modelling pipeline. The use of SMOTE as a part of the cross-validation system discouraged information leakage, and oversampling was limited to the training folds. After that, an 80/20 stratified train/test split was carried out to maintain proportions of classes in both subsets.

### Feature engineering

A set of derived features was designed to make the models expressive. The engineered variables included the measures of the intensity of the online activity, which included the number of total clicks, number of days active, and number of sessions, and the measures of assessment, such as mean score, cumulative grades, and the number of submissions. Temporal properties, especially time of registration and relative pacing with respect to the duration of modules, were also obtained. Together, these characteristics provided a more detailed insight into the behavioral and academic trends that succeeded and became inactive.

### Predictive modelling strategy

The predictive component of the study employed four supervised learning algorithms chosen for their complementary strengths and widespread acceptance in educational data mining:

- Logistic Regression, serving as the baseline linear classifier;
- Random Forest, offering robustness through aggregated decision trees;
- XGBoost, a gradient-boosting model well-known for accuracy and computational efficiency;
- Multilayer Perceptron (MLP), a feed-forward neural network capable of capturing complex, non-linear interactions.
- Each algorithm was integrated within a uniform processing pipeline constructed as:

[Preprocessing → SMOTE → Classifier]

This configuration ensured methodological consistency across all models, allowing for fair comparative analysis.

Model training and evaluation were carried out through Stratified 5-Fold Cross-Validation, preserving class distributions across folds while enabling reliability through repeated sampling.

### Ensemble learning extension

A bagging ensemble model was built to enhance predictive generalizability. Bagging generates several bootstrap samples of training data and trains different base learners on each sample. This last prediction of the ensemble is based on a combination of probabilities or majority voting and, in the process, eliminates variance and stabilizes the prediction. This can often result in such ensemble mechanisms being better than the individual models, particularly when there is noise or complicated interactions in the data.

### Explainability techniques

Two supplementary explainability frameworks were adopted to ensure transparency, a primary imperative of educational decision support systems. The disaggregation of global feature impact, explanation of patterns of contribution, and interpretation of how input variables moderated model prediction was used by Shapley Additive Explanation (SHAP). Local Interpretable Model-agnostic Explanations (LIME) provided instance-level explanations, explaining why a particular student is deemed to be at-risk or a success, by estimating the local behavior of the model on each instance. Combined, these methods can make the predictive system more interpretable so that the model outputs can be used to guide academic decision-making, as opposed to being black-box computation artifacts.

### Model development/predictive framework

This research work has compared several ML models, such as LR, RF, XGBoost, and MLP. Though the idea of ensemble learning (bagging and stacking) was taken into consideration, the ultimate predictive system is based on the proposed model, whose performance was found to be the best in all the evaluation measures. The method makes the choice of models clear and simplifies the reproducibility of the results.

## Results

It is a complete experimental process that assessed the predictive framework performance by means of stratified cross-validation of the training set, independent testing of a hidden hold-out set, and an interpretability evaluation using SHAP and LIME. In this section, the four baseline classifiers, as well as the ensemble approach, will be comparatively analyzed, and explanatory findings will be presented that can explain the behavior of the most effective model.

### Cross-validation performance

A stratified five-fold cross-validation was adopted to ensure that the class imbalance that is inherent to the outcome variable was sufficiently represented in each fold. They all had strong discriminative performance, and the measures of the performance were concentrated in a small range. However, differentiated rankings of them developed.

A total of 32,593 student records and multiple auxiliary datasets (assessments, VLE interactions, registrations, demographic attributes) were successfully merged and pre-processed to develop a predictive model for student academic success. After cleaning, encoding, and feature transformation, 49 predictors were retained, and the dataset was split into 80% training (n = 26,074) and 20% testing (n = 6519) while preserving class distribution.

Four baseline machine-learning models, including Logistic Regression (LR), Random Forest (RF), XGBoost, and Multi-Layer Perceptron (MLP) Neural Network, were evaluated using 5-fold cross-validation. Among them the proposed model achieved the highest mean ROC-AUC, outperforming all competitors with values consistently above 0.98, followed by the MLP model (ROC-AUC ≈ 0.979). The performance comparison confirmed XGBoost as the best overall classifier, with strong generalization across folds, as shown in Table 3.

**Table 3:** Cross-validation performance summary (K = 5).

Model	Mean ROC-AUC	Std. ROC-AUC
XGBoost	0.987948	0.001264
RF	0.986458	0.001353
LR	0.984126	0.001618
MLP	0.979606	0.001129

Table 3 presents that the cross-validation results clearly show that XGBoost achieved the highest and most stable performance, with the greatest mean ROC-AUC and lowest variability among all models. Overall, the ensemble-based methods (XGBoost and RF) consistently outpaced the linear and neural methods, indicating superior robustness for predicting student results.

This research work developed an ensemble ML model by using XG Boost, RF, LR & MLP integrated with XAI (SHAP & LIME) and achieved an accuracy of 95.04% by using statistical multiple Eqs. (1-8):

1 $${\mathrm{Accuracy}} = \frac{1}{n}\mathop \sum \limits_{i = 1}^{n} 1\left\{ {\hat{y}_{i} = y_{i} } \right\}.$$

Precision, Recall, and F1 per class ${\boldsymbol{c}}$:

2 $${\mathrm{Precision}}_{c} = \frac{{TP_{c} }}{{TP_{c} + FP_{c} }},$$

3 $${\mathrm{Recall}}_{c} = \frac{{TP_{c} }}{{TP_{c} + FN_{c} }},$$

4 $${\mathrm{F1}}_{c} = \frac{{2 \cdot {\mathrm{Precision}}_{c} \cdot {\mathrm{Recall}}_{c} }}{{{\mathrm{Precision}}_{c} + {\mathrm{Recall}}_{c} }}.$$

Macro- and Weighted-Average Metrics:

5 $${\mathrm{Metric}}_{{{\mathrm{macro}}}} = \frac{1}{C}\mathop \sum \limits_{c = 1}^{C} {\mathrm{Metric}}_{c} ,$$

6 $${\mathrm{Metric}}_{{{\mathrm{weighted}}}} = \frac{1}{{\mathop \sum \nolimits_{c} s_{c} }}\mathop \sum \limits_{c} s_{c} \cdot {\mathrm{Metric}}_{c} .$$

Confusion Matrix:

7 $$M_{ij} = \# \left\{ {k:y_{k} = i{\text{ and }}\hat{y}_{k} = j} \right\}.$$

ROC and AUC (One-vs-Rest):

8 $$AUC_{c} = AUC\left( {\{ \left( {y_{i,c} ,\hat{p}_{i,c} } \right)\}_{i = 1}^{n} } \right),y_{i,c} = 1\left\{ {y_{i} = c} \right\}.$$

As shown in Table 4, the cross-validation results demonstrate that XGBoost consistently delivers the strongest overall performance, achieving the highest scores across all major evaluation metrics. Ensemble methods, particularly XGBoost and RF, show superior robustness compared to LR and MLP, confirming their effectiveness for accurately predicting student success.

**Table 4:** Five-fold cross-validation performance of all models.

Model	Accuracy	Precision	Recall	F1-Score	ROC-AUC
XGBoost	0.950909	0.925579	0.974407	0.949338	0.987967
RF	0.949605	0.922610	0.975057	0.948096	0.986458
LR	0.942586	0.916440	0.966526	0.940804	0.984126
MLP	0.932155	0.921993	0.935490	0.928645	0.979606

### Test-set evaluation

The XGBoost classifier not only preserves but actually enhances its performance (at least in some cases) when used on the independent test set. The model attained an accuracy of 95.40, F1-score of 0.952, and ROC-AUC of 0.988, which highlights its high level of generalization. According to the classification report, the model is consistent in identifying both the academically good students and the students who are at risk.

Table 5 shows that the class-level results highlight the model’s strong discriminative ability, achieving high precision for at-risk students and excellent recall for successful learners. This balanced behavior indicates that the model reliably identifies true positives in both classes while minimizing misclassification, making it well-suited for early academic intervention and support systems.

**Table 5:** Precision and recall by class.

Class Label	Description	Precision	Recall
Class 1	Successful	0.93	0.98
Class 0	At-risk	0.98	0.93

**Figure 2:**
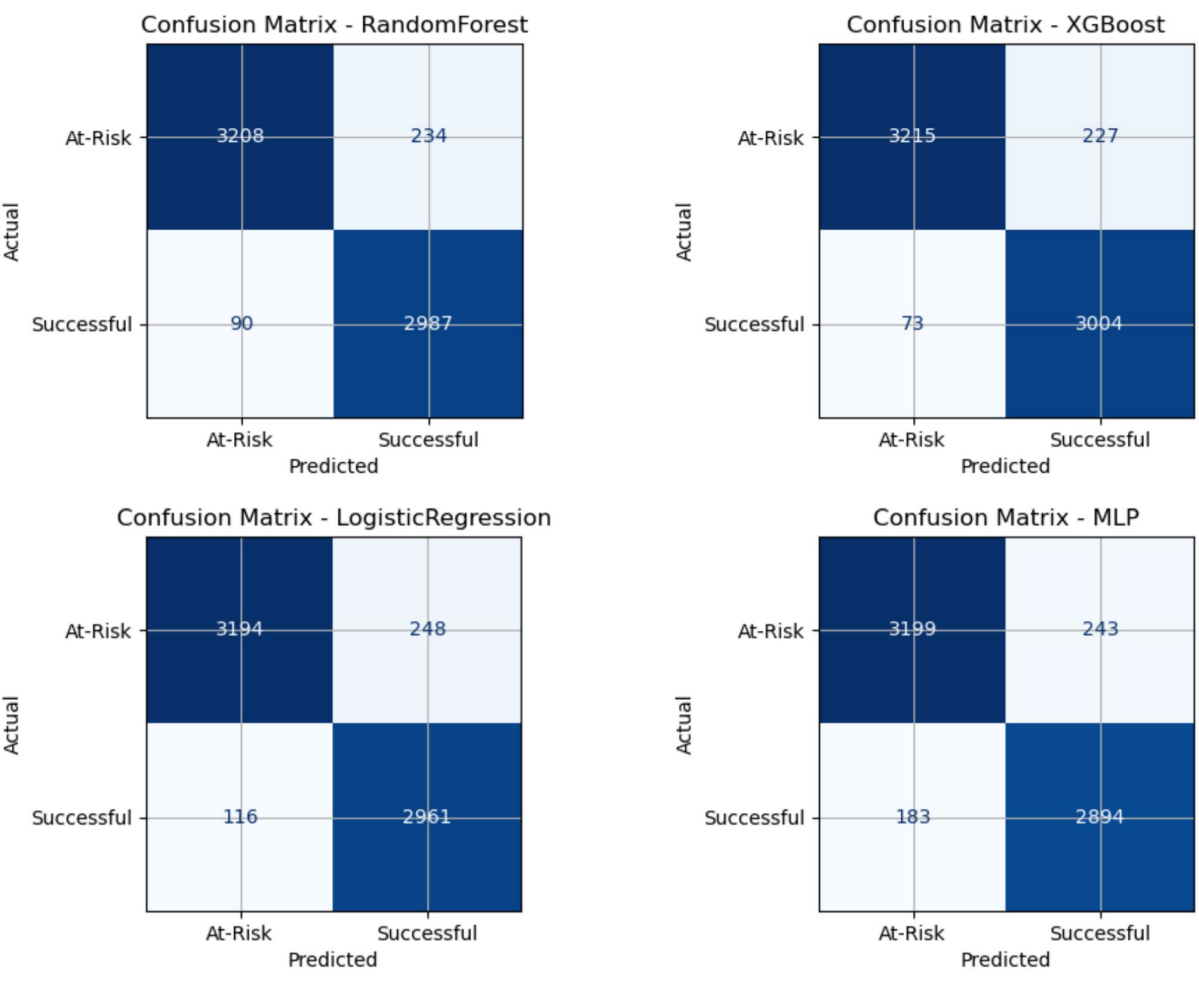
Confusion matrix of models.

Figure 2 presents the integrated confusion matrix results of all ML models. It is clearly seen that the XGBoost has made a few false identifications in a small percentage of the records, and it falsely identified 227 cases as positive and 73 cases as negative out of 6,519 cases, as compared to other models. These results affirm the fact that XGBoost can be well applied to the identification of learners who can undergo specific academic interventions at an early age.

Table 6 presents that the XGBoost model excels with the lowest false negatives (227), critical for early at-risk detection, while maintaining excellent overall accuracy, making it the optimal option for learning analytics deployment.

**Table 6:** Confusion matrix comparison across models.

Model	TP (At-Risk Correct)	FN (Missed At-Risk)	FP (False At-Risk)	TN (Success Correct)
XGBoost	3,225	227	73	3,004
MLP	3,199	243	183	2,894
RF	3,208	234	90	2,987
LR	3,194	248	116	2,961

**Figure 3:**
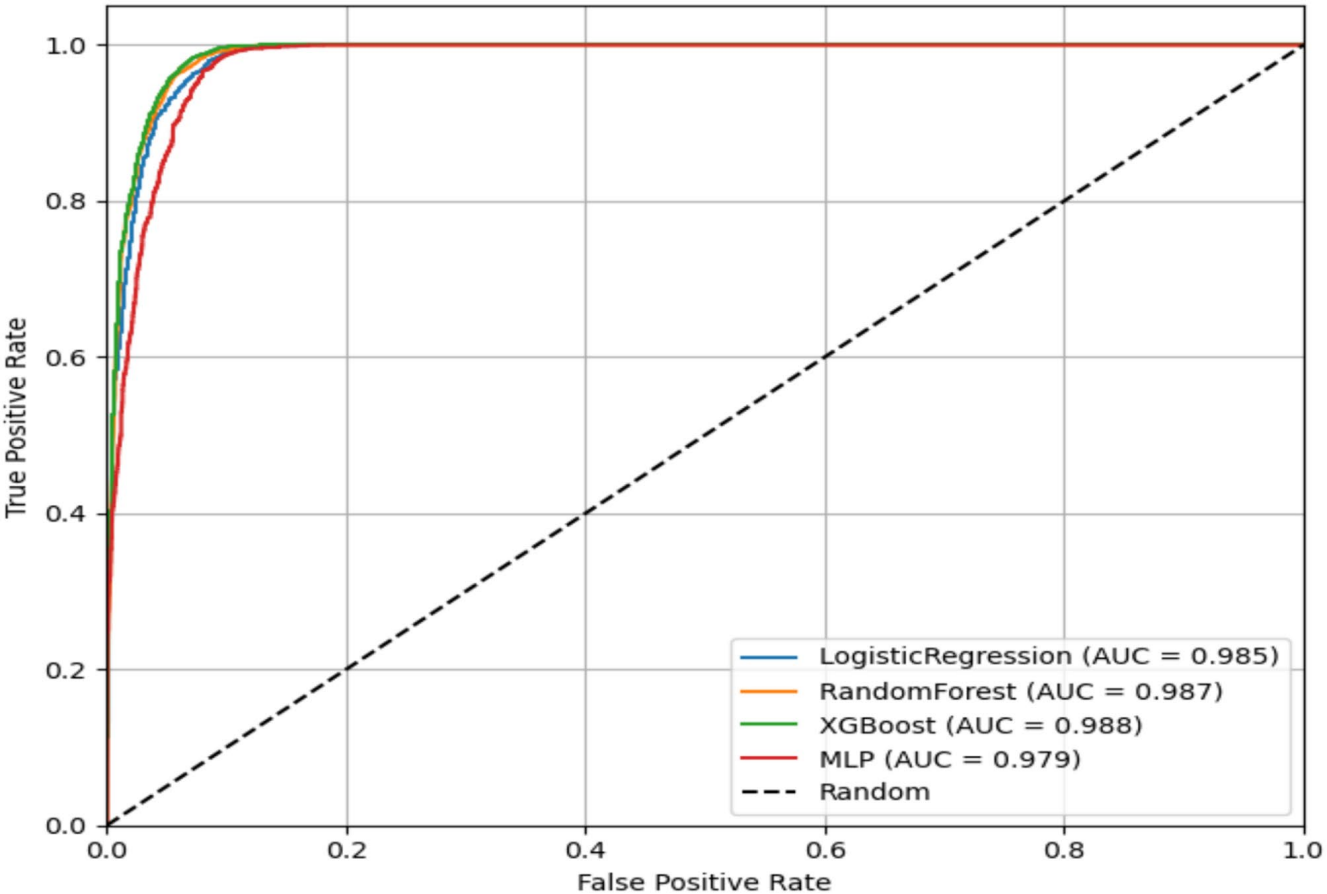
ROC curves—all models.

Figure 3 further confirms the comparative ROC curves and describes that all models achieve excellent discrimination (AUC 0.979–0.988) on the student success prediction task, with XGBoost and MLP slightly edging out RF and LR. Their tight clustering near the top-left corner reflects high sensitivity with minimal false positives, confirming the robustness of ensemble and neural approaches in learning analytics.

**Figure 4:**
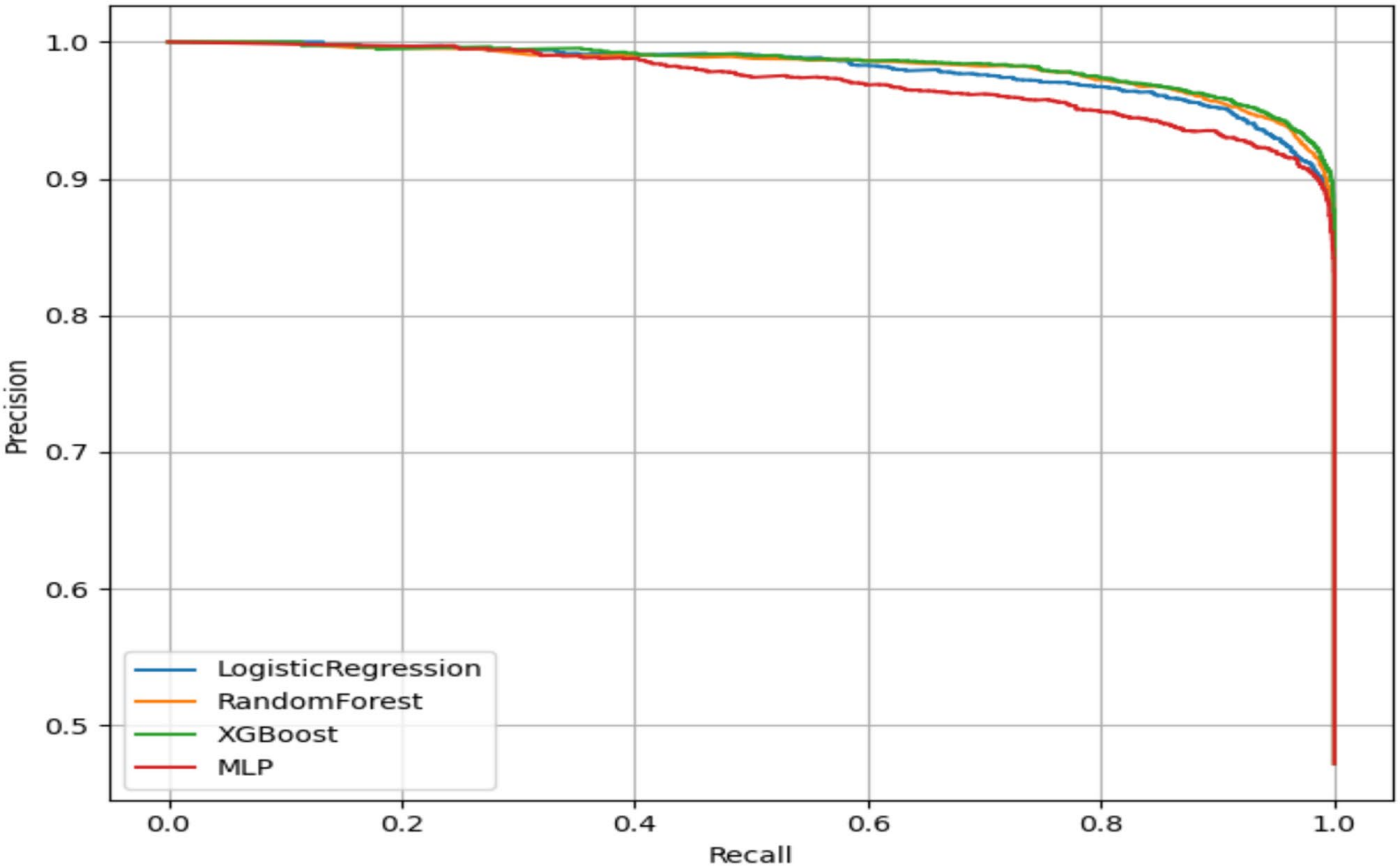
Precision-recall curves—all models.

Figure 4 presents the Precision-Recall curves for the four models on the student success prediction task. All models maintain high precision (>0.92) across nearly the full recall range, with XGBoost and MLP showing the most stable and highest curves, reflecting their superior ability to identify at-risk students with minimal false positives in this imbalanced learning analytics setting.

**Figure 5:**
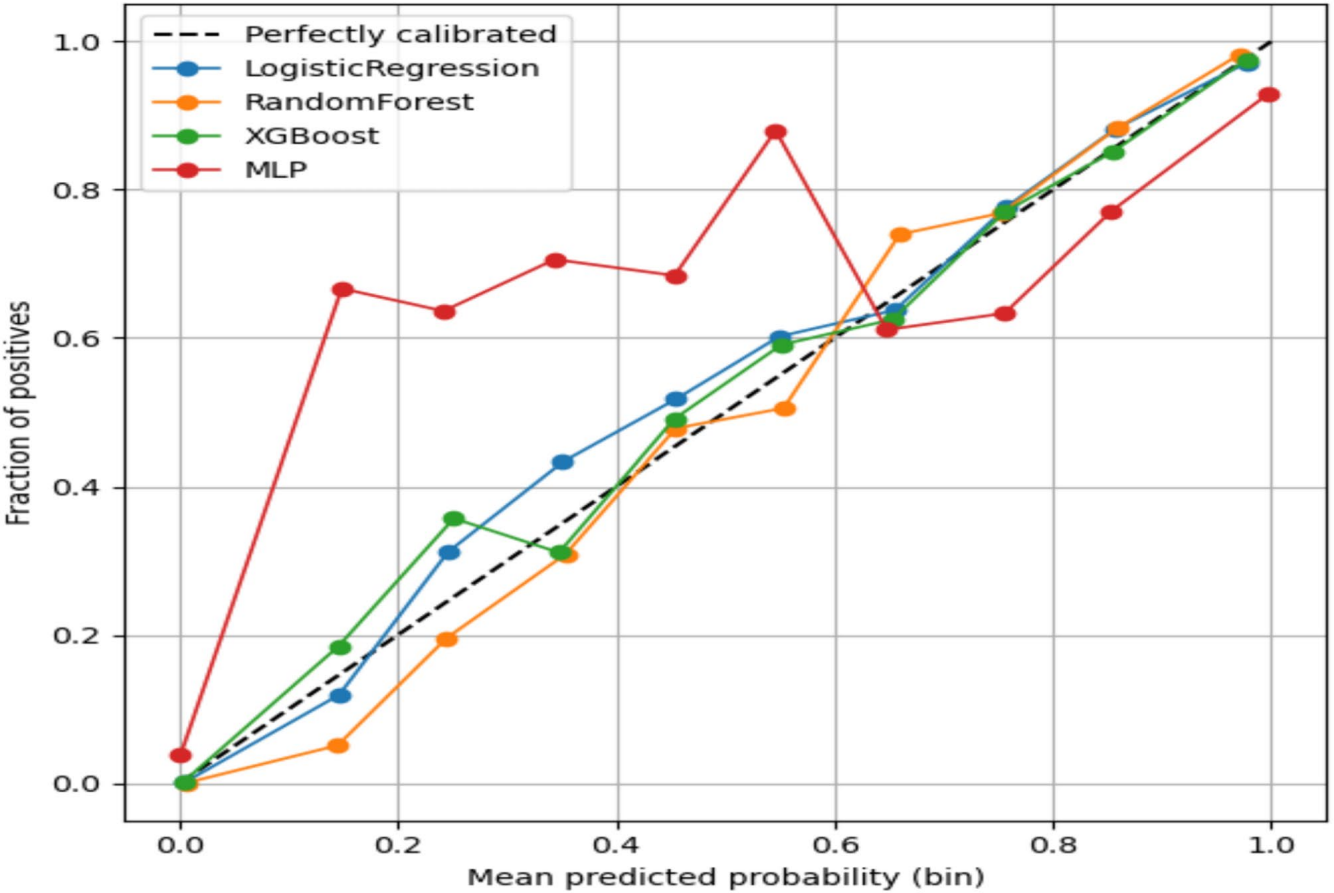
Calibration curves—comparison of models.

Figure 5 displays calibration curves comparing predicted probabilities against actual outcomes for the four models. XGBoost and RF closely track the diagonal (perfect calibration), while LR is slightly under-confident and MLP shows noticeable over-confidence in mid-to-high probability ranges. This highlights XGBoost’s superior reliability when probability outputs are employed for risk scoring in learning analytics interventions.

**Figure 6:**
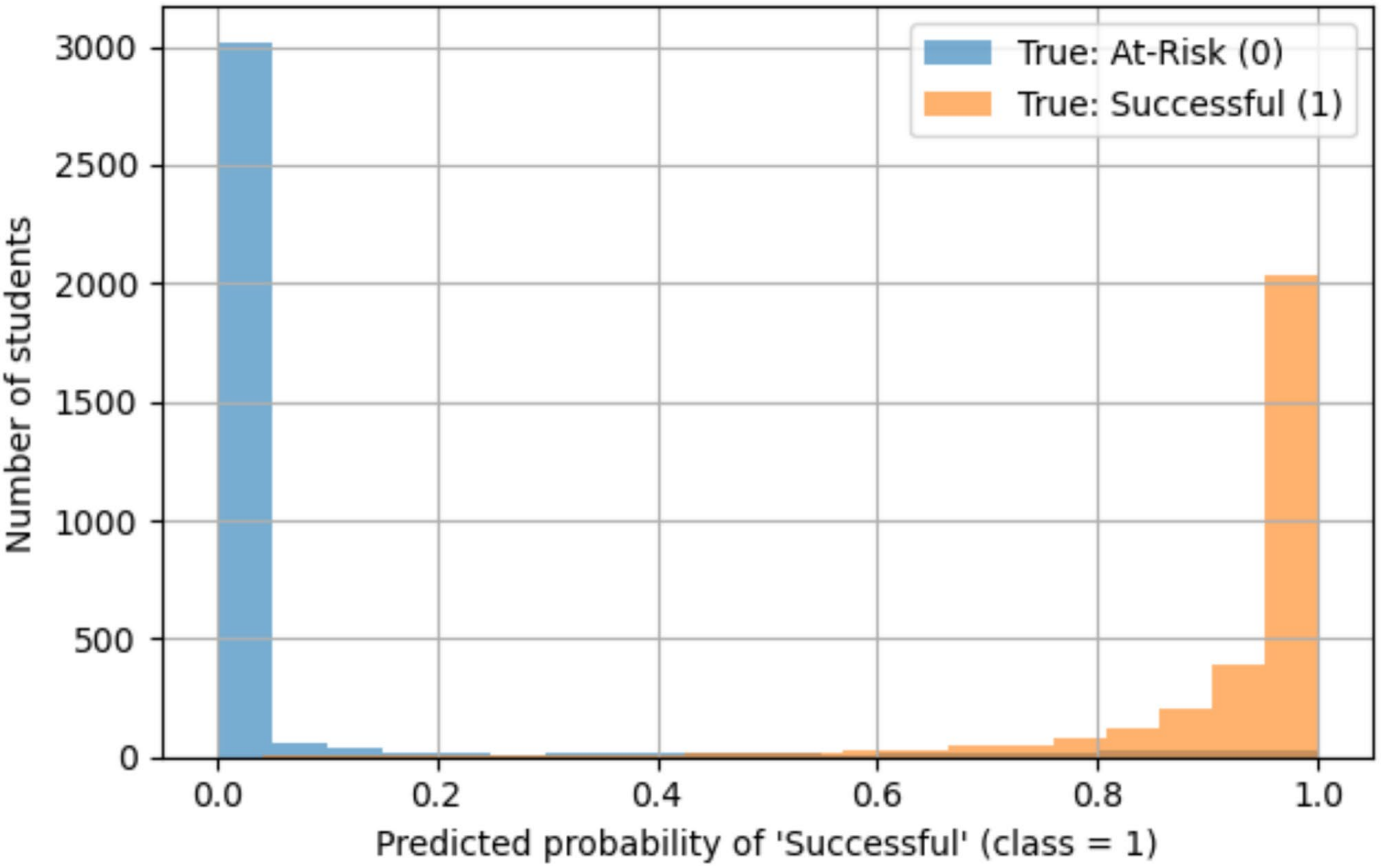
Predicted probability distribution by true class (XG Boost**).**

Figure 6 visualizes the distribution of predicted probabilities for the positive class (“Successful”, label = 1) on the test set. The clear bimodal pattern, with most “At-Risk” students confidently assigned low probabilities (<0.1) and “Successful” students pushed toward high probabilities (>0.8), demonstrates excellent probability separation and confirms the model’s strong discriminative power despite severe class imbalance.

**Figure 7:**
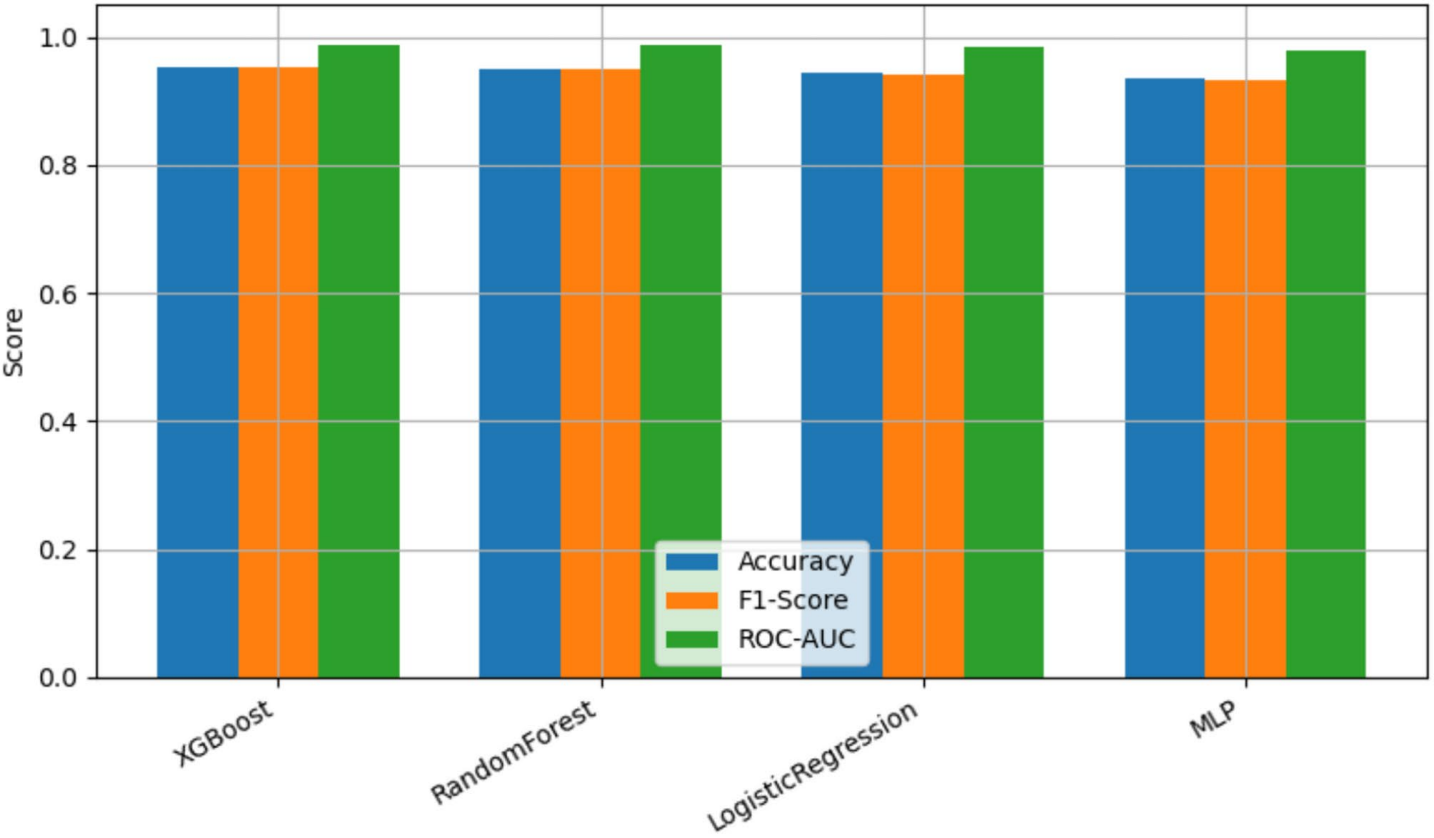
Performance comparison of models (test set).

Figure 7 compares Accuracy, F1-score, and ROC-AUC across the four models. While all achieve high scores (>0.94), XGBoost and MLP slightly outperform RF and LR on the more robust F1 and AUC metrics, confirming their superior balance of precision and recall in this highly imbalanced student success prediction task.

### Bagging ensemble performance

In order to establish whether ensemble aggregation might be additionally effective in increasing predictive strength or not, a Bagging model that used decision trees as the basis learners was fitted. The bagged ensemble showed a steady and good performance on folds with a mean cross-validated ROC-AUC of 0.9849, as shown in Table 6. The result is similar to the performance of the separate tree-based models. On the test set, the Bagging ensemble performed as highlighted in Table 7:

**Table 7:** Performance summary of bagging classifier (decision tree base).

Category	Metric	Value
5-Fold Cross-Validation Performance	Mean Accuracy	0.94876
	Mean Precision	0.92406
	Mean Recall	0.97132
	Mean F1-Score	0.94708
	Mean ROC-AUC	0.98497
Test set performance	Accuracy	0.95045
	Precision	0.92764
	Recall	0.97075
	F1-Score	0.94871

As shown in Table 7, despite the fact that its performance is sharply competitive, XGBoost remained superior to the ensemble regarding almost all the evaluation indicators, which once again proved its position as the leading model in the given study.

### SHAP-based global interpretability

The XGBoost model has been analyzed with SHAP in order to identify the most significant features that have a positive impact on the overall predictive results. Academic performance indicators, especially the total weighted score, the weighted average score, and the date of withdrawal/unregistration, were found to be the most significant predictors of the SHAP importance plot. Measures of behavioral engagement, including days active, the number of assessments, the number of clicks on different types of VLE resources, and module-specific categorical indicators, were also amongst the top-ranked measures. The SHAP bee swarm distribution demonstrated major trends: academic performance and longer engagement positively predicted future success, and delayed unregistering and significantly low cumulative performance were all major contributors to the at-risk categories. Dependence plots also demonstrated these relationships as the linear and monotonic changes with incremental gains in cumulative assessment performance were accompanied by a steep rise in the predicted possibility of academic success.

**Figure 8:**
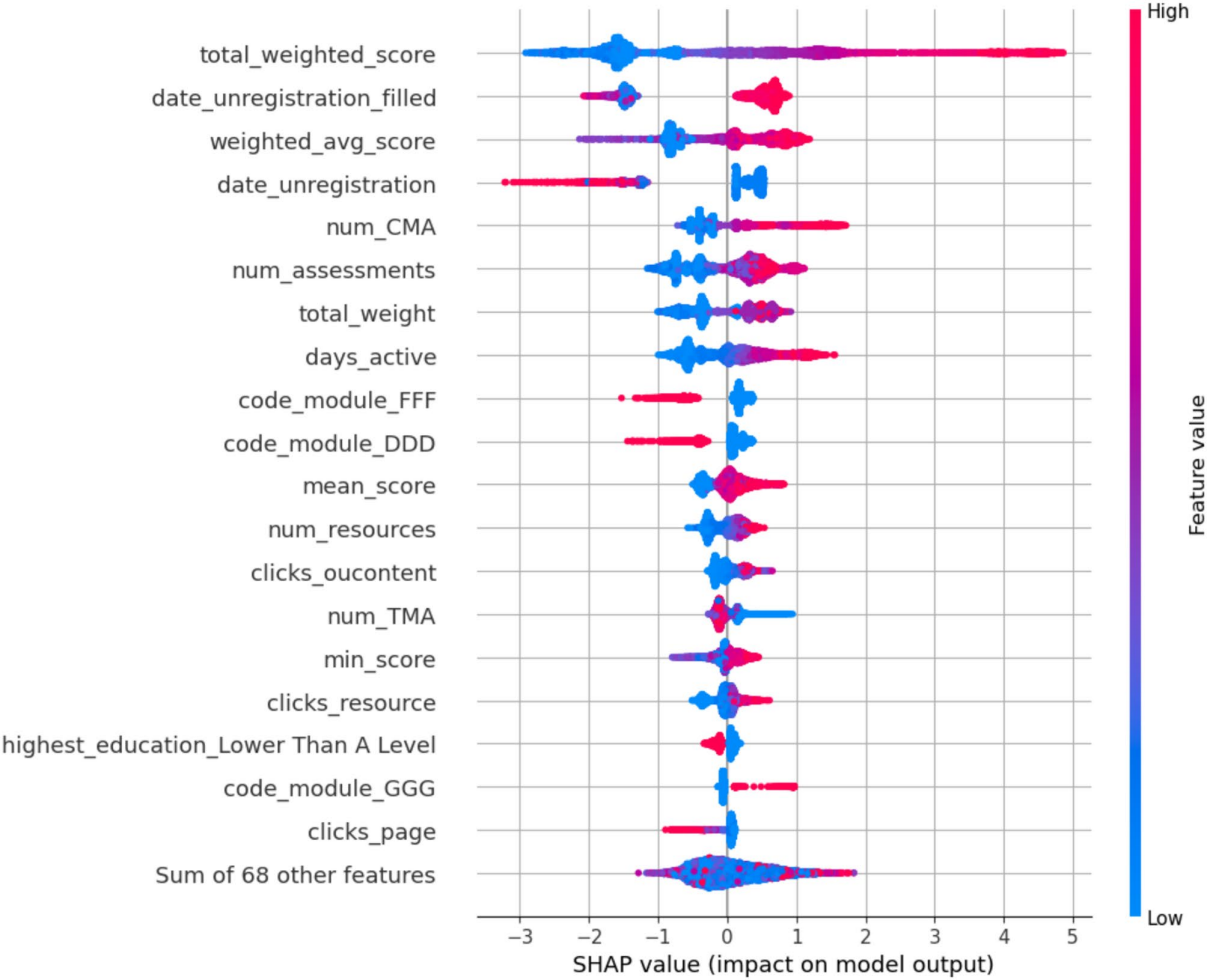
SHAP value (impact on model output).

Figure 8 presents the global SHAP summary plot for the XGBoost model, revealing the most influential predictors of student success. Early engagement features, such as total weighted score, average score, number of assessments submitted, and days active, dominate the top ranks with a strong positive impact, while late unregistration and lower prior education significantly increase dropout risk, providing clear, actionable insights for timely interventions.

**Figure 9:**
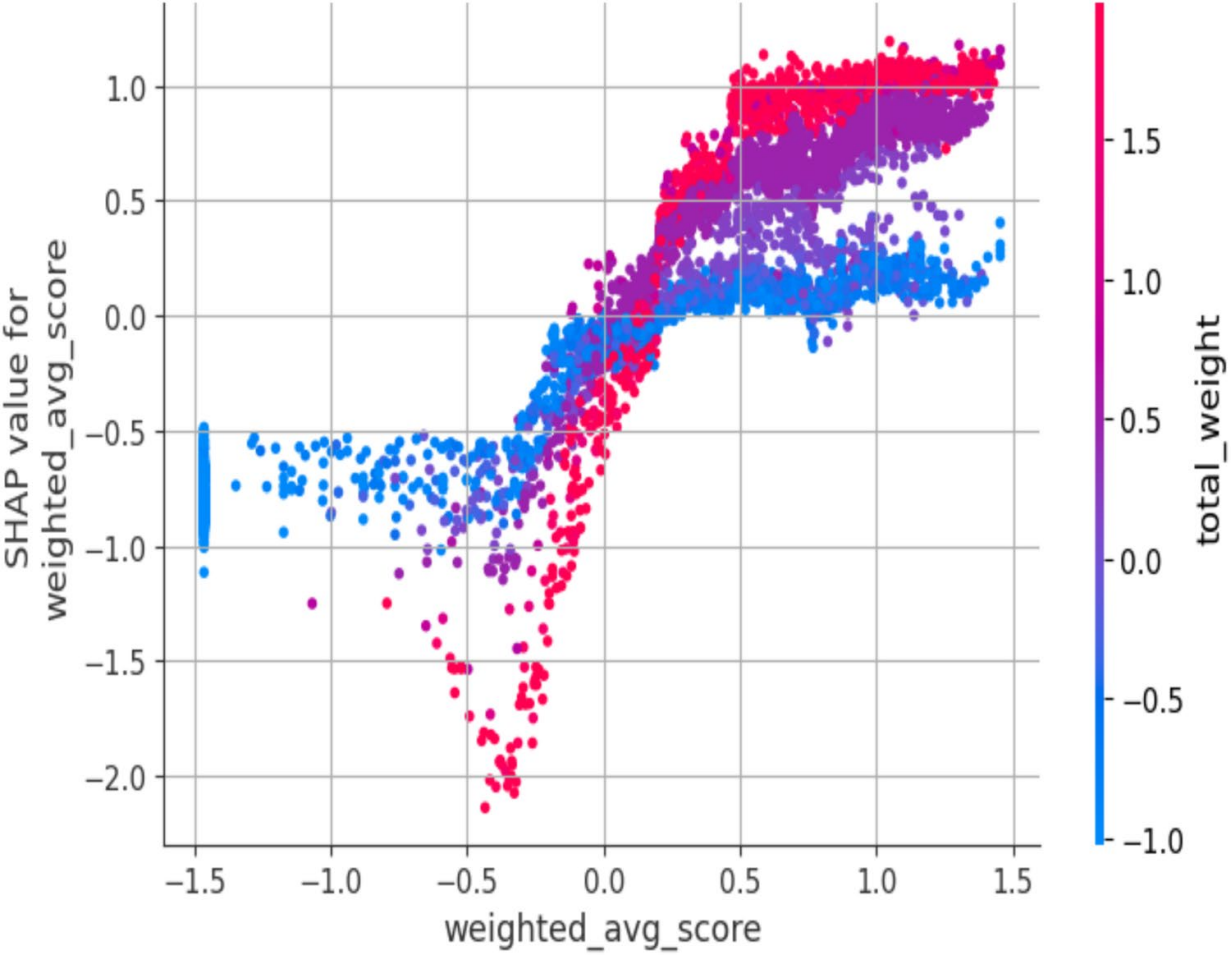
SHAP dependence plot—weighted_avg_score.

Figure 9 shows the SHAP dependence plot for the most important feature: weighted_avg_score. Higher average scores strongly push the model toward predicting success (positive SHAP values in red), while lower scores reliably increase the predicted risk of failure/withdrawal (negative SHAP in blue). The clear monotonic relationship confirms that early academic performance is the single strongest driver of student outcomes in the model.

**Figure 10:**
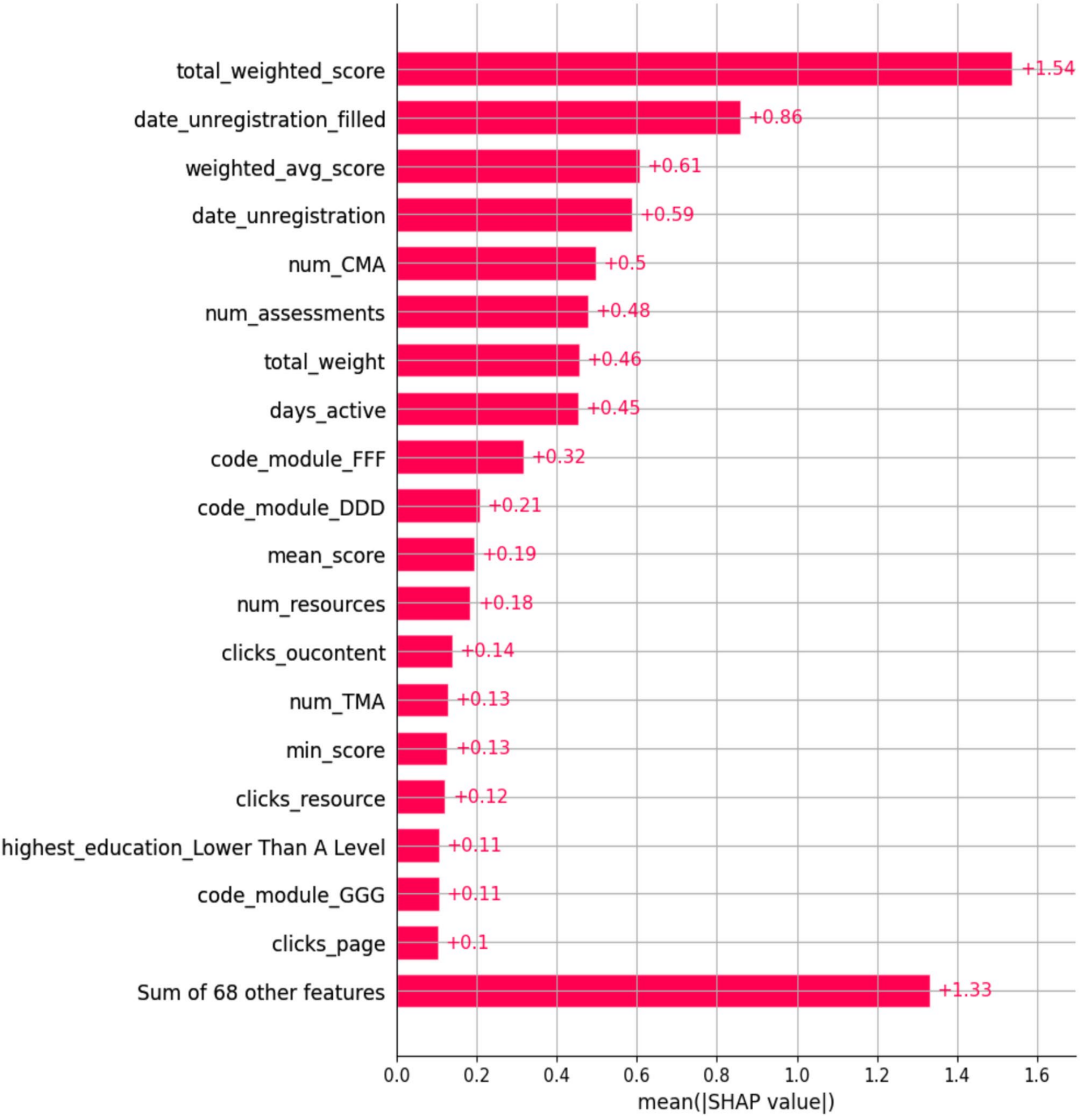
Mean (|SHAP value|).

Figure 10 displays the mean absolute SHAP values for the top features in the XGBoost model. Total weighted score dominates with the highest impact (+1.54), followed by early unregistration date and weighted average score, confirming that strong early academic performance and continued enrollment are the strongest protective factors against dropout, while late withdrawal is a major risk signal. This provides clear, evidence-based targets for proactive student support.

### Local interpretability through LIME

To supplement the global interpretative framework, the local interpretable model-agnostic explanations (LIME) methodology was used to explain predictions at the granular level, that is, at the individual level. Explanatory results were obtained on various representative test cases that belong to the successful and at-risk groups.

In the whole sample of the cases assessed, LIME demonstrated regular emphasis on the fundamental performance indicators, such as total weighted score, weighted average, and the early withdrawal behaviors, as the main determinants of the classification results. The secondary influences included contextual factors like module code, discrete resource interaction patterns, and days active on the platform.

Amongst the student group projected as at-risk, the combinations of significantly poor weighted scores, early withdrawal events, and significantly decreased engagement were predicted by LIME as among the strongest drivers. On the other hand, when the success level of students whose success status was correctly forecasted, it recurring high-engagement and strong-assessment performance was identified as a salient positive contribution.

**Figure 11:**
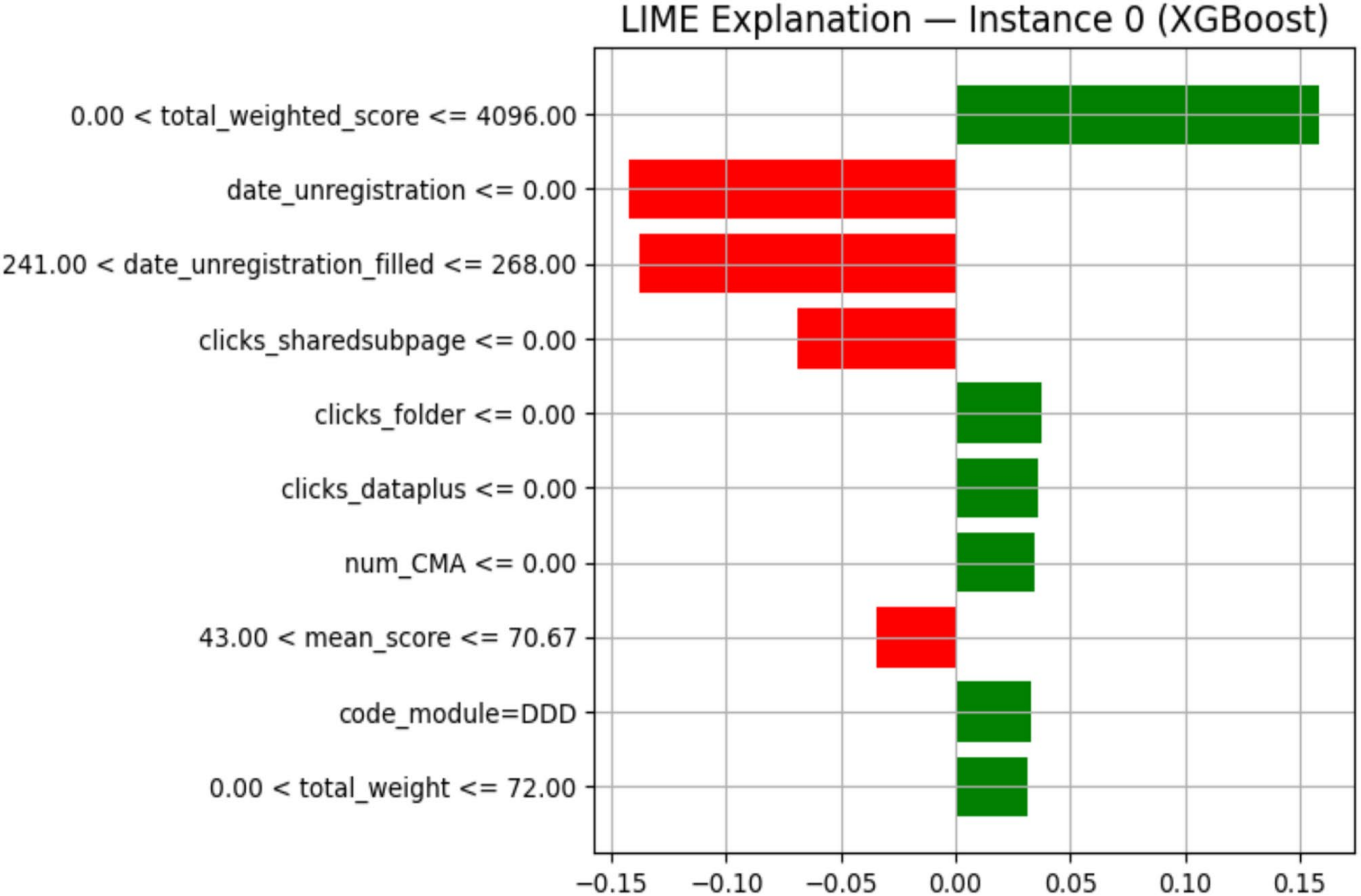
LIME explanation—instance 0 (XGBoost).

Figure 11 shows a LIME explanation for a correctly predicted “At-Risk” student (Instance 0). The strongest driver pushing toward failure is an extremely low total_weighted_score (<4096), followed by early unregistration and no clicks on key resources. Conversely, being enrolled in module DDD and some minimal engagement slightly mitigates the risk, but not enough to offset the dominant early academic warning signals.

The high level of concordance observed between the SHAP and the LIME results supports the accuracy of the predictive model and increases the trust in the clarity of the predictive model’s decision-making processes.

As shown in Table 8, the strongest model was XGBoost with an accuracy of 0.95398, macro-average F1-score of 0.95244, and ROC-AUC 0.98834. Class-level measures and confusion matrix show that there is balanced performance between At-Risk students and Successful ones, and that there is little misclassification. The strong precision-recall patterns and high true-positive rates further confirm that the model is both robust and effective for early identification of at-risk learners in educational environments.

**Table 8:** Comprehensive evaluation summary of the XGBoost Model (Best Model).

Section	Metric/Description	Value
Model Selection	Best Performing Model (Based on CV ROC-AUC)	XGBoost
Overall Test Performance	Accuracy	0.95398
	F1-Score	0.95244
	ROC-AUC	0.98834
Class-Level Performance	Class: At-Risk (0)	
	Precision	0.98
	Recall	0.93
	F1-Score	0.96
	Support	3442
	Class: Successful (1)	
	Precision	0.93
	Recall	0.98
	F1-Score	0.95
	Support	3077
Aggregated Metrics	Overall Accuracy	0.95
	Macro-Average F1-Score	0.95
	Weighted-Average F1-Score	0.95
Confusion Matrix	True Negative (Not Successful → Not Successful)	3215
	False Positive (Not Successful → Successful)	227
	False Negative (Successful → Not Successful)	73
	True Positive (Successful → Successful)	3004

Table 9 compares earlier models with the proposed ensemble ML + XAI approach, showing that previous methods achieved accuracies between 83–93%, with varying use of SHAP.

**Table 9:** Comparison of the proposed model with previous models.

References	Model Used	Dataset	XAI Integrated Model	Accuracy (%)	Miss Rate (%)
^25^	Random Forest, XGBoost, DNN	OULAD	No	85.0	15
^27^	Federated Learning	OULAD	No	85.0	15
^29^	LSTM + SHAP	OULAD	Yes [SHAP]	92.88	7.12
^30^	LSTM, CNN, RNN	OULAD	No	83.41	16.59
^29,31^	Bi-LSTM + SHAP	OULAD	Yes [SHAP]	88.23	11.77
	Proposed Approach (Ensemble ML + XAI)	OULAD	Yes [SHAP & LIME]	95.04	4.96

The proposed model performs best overall, reaching 95.04% accuracy and the lowest miss rate (4.96%), demonstrating clear improvement.

**Figure 12:**
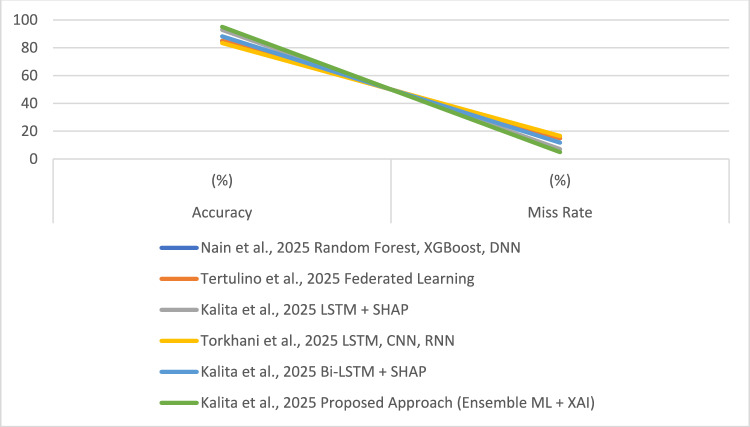
Graphical Representations of the proposed model performance with the previous models.

Figure 12 compares several studies by presenting each model’s high accuracy and corresponding low miss rate, highlighting overall strong performance across techniques. It also shows that the proposed ensemble ML + XAI approach performs competitively, aligning closely with or slightly outperforming previous models.

## Conclusion

This study developed an interpretable predictive framework to identify academically at-risk students in large-scale digital learning environments. By integrating seven datasets, including demographics, registration patterns, assessment history, and VLE engagement, complex learning behaviors were effectively modelled using modern ensembling machine-learning techniques. Among the models tested, XGBoost performed best, achieving 95.1% accuracy, 0.926 precision, 0.974 recall, 0.949 F1-score, and 0.988 ROC-AUC, while Random Forest, Logistic Regression, and MLP showed slightly lower but competitive performance, confirming the strength of tree-based ensembles for early-warning prediction. The framework’s interpretability, enabled through SHAP and LIME, revealed that cumulative assessments, withdrawal indicators, participation timing, and VLE interaction intensity were key predictors, consistent with learning-engagement theories. These results highlight machine learning’s potential to support timely, data-driven academic interventions. Future work may explore temporal deep-learning models, multimodal features, causal inference, and fairness-aware evaluation to improve predictive reliability and ethical grounding.

The proposed framework also has chances to be adapted to the new educational systems. To this extent, transfer learning methods can ensure that the model can be optimized using data from other institutions, thus becoming useful in diverse academic settings. Besides, an integrated learning analytics system, which combines predictive modelling with institutional dashboards and real-time monitoring tools, would help to deploy it at scale and enable data-driven academic decision-making on the organizational level.

### Limitations and future work

Despite the strong predictive capability and explanatory quality of the suggested framework, there are still a few substantive limitations. The present research has been based on the OULAD dataset that is a particular online learning setting and could be not generalized to other learning settings. The differences in institutional settings, student behavior, and structures of the curricula could affect model performance when implemented in other settings. The model utilizes a fixed paradigm and ignores the dynamics of behavioral changes, which can better be represented by sequential or time deep-learning architectures. Although SMOTE has been used to address the problem of class imbalance, the synthetic oversampling method can inject perturbations, which supports the need for more resilient imbalance-management techniques. Moreover, the feature-engineering pipeline does not use some sophisticated methods, such as graph-based interactions, sequence embeddings, and text-based inferences based on student discussion forums.

Future studies should therefore include time-sensitive design (LSTMs), GRUs, or Transformers, include multimodal learning analytics modalities to learn more about behavior, and use causal-inference infrastructure to query the impact of particular learning behaviors. The further enhancement of the relevance and reliability of the proposed system will be a development of real-time instructor dashboards, an investigation of reinforcement-learning or meta-learning paradigms of adaptive interventions, and extensive fairness and ethical assessments.

The future research work also concentrates on exploring the effects of the inclusion of non-academic conditions, such as stress-related, psychological, physiological, and environmental factors, to create more context-relevant, comprehensive, and practically functional predictive models that can more easily capture the actual real-world performance among students.

## Data Availability

The original contributions presented in the study are included in the article; further inquiries can be directed to the corresponding authors.

## Code availability

The code used in this study is publicly available and can be accessed via GitHub at: https://github.com/EssaAlmazroei/Test/blob/master/Code.ipynb. The repository contains the implementation of data preprocessing, model development, and analysis procedures used in this research.
